# Can eggshells indicate stressor exposure in free-range laying hens?

**DOI:** 10.1017/awf.2024.46

**Published:** 2024-11-20

**Authors:** Helen E Gray, Emma L Malcolm, Katherine Herborn, David Armstrong, Jessica E Martin, Lucy Asher

**Affiliations:** 1 School of Natural and Environmental Sciences, Newcastle University NE1 7RU, UK; 2 School of Biological and Marine Sciences, University of Plymouth PL4 8AA, UK

**Keywords:** Animal welfare, non-invasive, shell colour, shell quality, shell strength, shell texture

## Abstract

Finding effective ways to monitor laying hen welfare is challenging as UK flock sizes can reach 16,000 birds. Eggs provide potential for welfare monitoring, as they are a daily output with previous evidence of links to stress. We explored the associations between stressors and eggs using two complementary studies. In Study 1, hens experienced social or heat stressors and eggs were scored daily for defects in shell characteristics. All eggs were scored on a three-point scale: 1 (no defect); 2 (minor defects); or 3 (unsuitable for whole egg sale in the UK). Texture defects were higher after stress treatments and were explored further as a promising proxy measure of welfare. In Study 2, eggshell texture from five commercial flocks was scored before versus at the onset of an avian influenza-enforced indoor housing, and scores were correlated with industry data for egg quality. Eggs were more likely to have texture defects after the enforced indoor housing, and manually scored texture correlated significantly with shell strength and shell colour during automated grading. Shell strength was weaker immediately after the enforced indoor housing and eggs were darker. We suggest that eggshell texture could be a useful addition to assessing changes or stresses in a hen’s environment for both research and commercial purposes, but further validation is needed to understand the generalisability of these results to other stressors. Additionally, data already collected in factories, such as shell strength and colour, may provide information on stress and could be valorised for understanding hen welfare.

## Introduction

In the UK, free-range hens spend around 60 weeks of their lives producing eggs. During this time, they may experience a range of social stressors including feather pecking (55% of UK flocks; Rodenburg et al. 2013), injurious behaviour (Krause *et al.* 2011), resource competition (Hunniford *et al.* 2014) and piling (Gray *et al.* 2020). In addition, environmental stressors, including heat stress (above 30°C; Mignon-Grasteau et al. 2015), predator exposure (Moberly et al. 2004), and the routine disturbances of an industrial farm context, such as stockperson disturbances, alterations to lighting and heating schedules, loud noise and litter changes. Sudden or threatening events trigger a cascade of physiological changes that enable animals to react quickly and cope with immediate challenges. However, recurrent acute stressors or stress exposure across modalities can result in chronic stress (Campderrich et al. 2019; Campbell 2023). This has wide-ranging impacts upon physiological function and health, including disease progression (Li et al. 2013; Golbidi et al. 2015) and metabolic traits (Malik & Spencer 2019) such as energy expenditure and fat deposition. For producers to minimise the risk of these downstream cumulative impacts on welfare and productivity, there is a need for continuous, tractable, holistic markers of overall function, to isolate the impacts of stress. Better or poorer function than expected on a set date (given known or routinely monitored stressors) could help pinpoint welfare challenges that are unknown, emerging or not easily observed. Moreover, this could provide evidence to evaluate strategies intended to increase flock resilience to stress, such as targeted breeding programmes (Berghof *et al.* 2019) and in early life husbandry (Campderrich et al. 2019). As eggs are a daily output of commercial laying farms, and their formation is governed by multiple physiological processes, this paper explores the potential for eggshell quality to provide this proxy measure of stress in laying hens.

Egg collection and assessment is increasingly automated, so there is great potential to include eggshell characteristics within routine, daily monitoring. Each hen lays only one egg per day, so this approach offers scope uniquely to collect individual-level data and monitor intrapopulation variability in large flocks (in the UK, up to 16,000 birds). Research on eggshell characteristics has generally focused on the strength or thickness of the shell (Dawkins et al. 2004; Bain 2005; Mertens et al. 2006; Solomon 2010) as this has the largest financial impact on egg production; eggs with cracked or broken shells may not be sold. However, eggshell characteristics may also refer to other abnormalities including rough shell texture (Wengerska *et al.* 2023), wrinkling or creasing of the shell surface (Wengerska *et al.* 2023), an uneven or unexpected colour (Hughes et al. 1986), and unusual shaped eggs (Hughes *et al.* 1986). Many of these abnormalities can lead to eggs being downgraded to Grade B (colloquially known as ‘seconds’), which is associated with a lower price per egg.

Stress-induced elevation of corticosterone can alter sex steroid secretion governing egg production (for a review, see Oguntunji & Alabi 2010). Artificially elevating baseline corticosterone over days to simulate different intensities of chronic stress can lead hens to reduce or cease egg production (Etches *et al.* 1984). Within days, individual stressful events may alter eggshell structure directly via the actions of adrenaline on cuticle formation and timing of oviposition, altering time for calcium deposition (Sykes 1955; Solomon et al. 1987). Acute stressors may also indirectly affect eggshell characteristics through behavioural ‘fight-or-flight’ responses that physically disturb birds during shell formation or laying. Indeed, calcium deposition is observed in hens that are disturbed or are more behaviourally fearful (Hughes *et al.* 1986; Mills *et al.* 1991). There appears to be some consistent effect of different stressor modalities on egg formation. For example, commercially relevant stressors such as disturbance, simulated feather pecking and high densities are all associated with paler eggs in brown-egged strains and with extraneous calcium deposition (for a review, see Samiullah et al. 2015). However, egg formation is a complex physiological process spanning around 24–26 h and other welfare challenges, that may also cause stress, can alter eggshell characteristics via different pathways. Heat stress, specifically, has been widely studied in relation to eggs as it is known to negatively impact production (Oguntunji & Alabi 2010; Mignon-Grasteau *et al.* 2015). Reductions in shell thickness under heat stress are caused in part by a reduction in the calcium carrying capacity of the blood due to alkalosis that occurs with panting (Marder & Arad 1989). Whereas diseases including infectious bronchitis and Newcastle disease also cause lightening but via disruption to cellular mechanisms for pigment deposition or damage to the oviduct (for a review, see Samiullah et al. 2015). Other factors affecting egg characteristics that may involve different pathways include age-related change and nutrition including provision of probiotics (for a review, see Samiullah et al. 2015). It will be important therefore to determine whether changes in eggshell characteristics are of consistent direction and provide a domain-general marker of stress exposure or can potentially also differentiate specific welfare challenges. It is interesting to note that much of the research linking stress and eggshells is not recent and yet these results have not been fully utilised by the industry. It could be that now is an opportune time to re-examine eggshells as a potential indicator of stress with recent technological advances being able to support automated detection of eggshell quality (So et al. 2022; Chen et al. 2023) and a recognised need for animal-based outcome measures of animal welfare for precision livestock technologies (Rowe *et al.* 2019; Tuyttens *et al.* 2022).

In this paper, we present two complementary studies investigating the potential for eggshell quality to be used as an indicator of stress. In Study 1, we conducted an experiment in which stress was directly manipulated in laying hens and eggshell quality was manually scored. Two different manipulations of stress were applied: (i) isolation of chickens from conspecifics as a social stressor; and (ii) a mild heat stress as a physiological stressor. For Study 2, we made use of a country-wide enforced indoor housing in 2022 which is a biosecurity measure against Avian Influenza in which access to the outdoor range was withdrawn for free-range chickens. Specifically, we assessed the immediate, day 1, effect of the containment. This sudden change in environment and reduction in choice and range is likely associated with dynamic changes in general flock behaviour to adjust, and therefore is a potential stressor. Furthermore, based on hens’ behaviour in choice experiments, they actively choose to be outside/have access to range over access to food or companions (Dawkins 1977), therefore loss of this access may be stressful. In addition, hens with access to a range area have been found to have lower measures of stress than those without this access (Campo *et al.* 2008). We collected eggs and data on shell defects from a single egg-packing factory (processes approximately 10 million eggs per week) before and after the enforced indoor housing was imposed.

We formed two hypotheses regarding the association between eggshells and stressor exposure:

- H1 (Study 1): Exposure to social isolation or high temperature will be associated with poorer egg quality parameters as measured by shell texture, shell-less eggs, soft shells, wrinkles and cracks.
  - The two treatments will be compared to investigate whether changes in shells are a domain-general indicator, or stressor-specific.
- H2 (Study 2): Eggshell qualities found to be associated with stress in Study 1 would be higher after the enforced indoor housing than before.

We did not make specific hypotheses about data on egg defects from the egg-packing factory before and after the enforced indoor housing and considered this analysis exploratory, designed to create new hypotheses which can be tested in future research.

### Ethical approval

The experiment was approved by the Newcastle University Animal Welfare Ethical Review Board (AWERB), reference number 873. The pilot study which preceded the current experiment and our hypotheses were pre-registered to follow an Open Science approach (https://doi.org/10.17605/OSF.IO/3F8VZ). Data from eggs of commercially housed laying hens (both manually collected and commercially available) were collected opportunistically due to the occurrence of a country-wide biosecurity measure which was a presumed stressor.

## Study 1

## Study animals

For Study 1, two hundred and forty 59-week-old Shaver Brown hens were collected from a commercial laying farm in Cumbria. This age is beyond peak production (~25–31 weeks), but egg production is still expected to be high at this age. One hundred and twenty hens were randomly taken from one shed, and 120 from a second shed. The sheds were identical in size, flock age and stocking density (8.6 birds m^–2^). Hens were caught by experienced stockpersons. The chickens were transported in crates of different sizes in a ventilated vehicle to Newcastle University; twenty-one smaller crates (85 × 50 × 31 cm; length × width × height) each contained nine hens; four larger crates (95 × 57 × 24 cm) held ten hens, and one contained eleven. The journey time was approximately 120 min, with a distance of 85 miles and the temperature and relative humidity on the day were 15°C and 80–90%, respectively.

**Figure 1.**
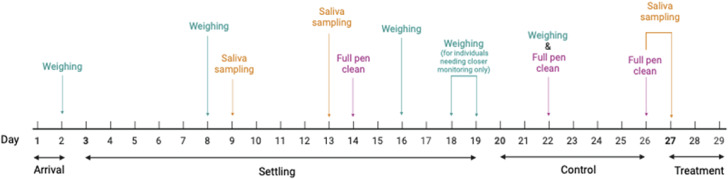
Timeline of events for each stage of the experiment. Weighing refers to the weighing of laying hens. Eggs were scored during the control and treatment phases. Note that egg scoring is indicative of the events of the previous day, such that those eggs laid on day 27 reflect the control conditions of day 26. Saliva samples did not undergo any further use in this study but are included for completeness. See text for further details. Figure created with BioRender.com.

The timeline of study events, husbandry and routine weighing can be seen in Figure 1. On arrival, the order in which crates were unloaded from the vehicle designated assignment to specific rooms in the facility (e.g. first crate to room one, second crate to room two). Crates of hens in each room were then split between the pens until each pen contained 15 hens. Although this method was not completely random, it was an efficient way of ensuring a distribution of hens from the two flocks across pens without the hens spending longer than necessary in the transport crates. The following day hens were individually weighed and identified with a unique leg band (plastic flatband split rings; Avian ID, UK; avianid.co.uk) on the left leg.

Focal hens, from which saliva samples would be taken (see Study 1, *Saliva sampling*), were selected on Day 7. These data were not further used in the present study as this work is ongoing and therefore is only described in *Saliva sampling* for completeness of the stressor experienced and for scientific transparency and openness. Salivary corticosterone is yet to be validated for chickens and therefore samples from this study are being combined as part of a larger body of work, including a subsequent study comparing salivary and blood corticosterone currently under analysis. As expected, after transportation and adjustment to new housing, new feeders and smaller group sizes, mean (± SD) body mass of the hens reported a loss: 4.00 (± 5.14)%. To guard against selecting hens needing a larger weight increase to regain condition, focal hens were selected from those which had lost less than 5% of their bodyweight. Although this was not a selection from the total pool of hens, this method allowed for random selection within a subgroup of hens whilst prioritising animal welfare.

In line with our ethical documentation, if any hen’s welfare was suspected of being compromised through conspecific bullying or other illness/poor condition, the affected hen was assessed by the Named Animal Care and Welfare Officer. If deemed necessary, the hen was removed from the study and placed into one of two ‘sick rooms.’ These rooms measured 4.0 × 3.2 m (length × width) and contained deep shavings, bell feeders, bell drinkers and enrichment (CDs, hanging ropes, perching). In place of fixed nest-boxes, these rooms had pet carriers. Sick rooms were checked a minimum of twice per day. In total, 16 hens were moved to the sick rooms prior to the start of the treatment period due to poor feather cover and/or weight loss, and did not rejoin the study. One hen’s condition remained poor as she was the subject of vent pecking even after interventions to minimise this behaviour, and this individual was euthanased three days after transfer to the sick room. The remaining 15 hens were rehomed.

## Materials and methods

### Housing

**Figure 2.**
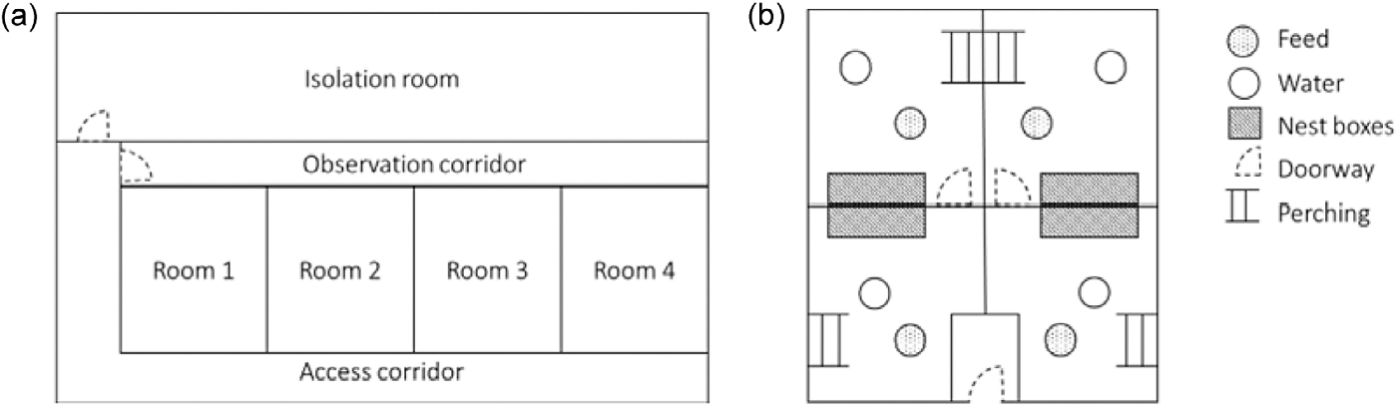
Plan of the experimental set-up used for the laying hens in Study 1 showing (a) holding rooms and treatment rooms and (b) the layout of an individual holding room.

Hens were housed in one of four rooms, each containing four pens (Figure 2[a]). Rooms were 7 × 4 m and each pen measured approximately 2 × 3 m. All pens contained six nest-boxes (Gaun, Murcia, Spain), a bell drinker and bell feeder, perching, deep shavings to cover the floor area, a hanging enrichment (tub with shiny bolts inside), CDs and cardboard (basic layout shown in Figure 2[b]). All hens had *ad libitum* water and feed (layer mash; Carrs Billington, Morpeth, UK), and daily access to mixed grit. The target temperature of the rooms was set to 18.5°C and light:dark cycle was 14:10 (to mimic the sheds of origin), with lights on at 0500h and off at 1900h.

### Settling phase

On settling days, the rooms were checked hourly between 0800 and 1100h and the number of eggs produced in each room was noted during collection. This allowed us to estimate laying time of the hens such that we could time our treatments to coincide with the shell formation of the next egg. Feed was topped up and fresh water provided in the mornings.

### Control

On control days, eggs were collected throughout the morning and standard husbandry continued (feed and water changes, and mucking out), but no other daily protocols were conducted. On day 26, baseline saliva samples were taken (see *Saliva sampling*) and a routine pen clean was carried out.

### Treatment

Treatments began on Day 27 and finished on Day 29. We had initially planned for six days of treatment but, unfortunately, this was cut short due to extreme weather conditions in the UK (Storm Arwen) making working conditions unsafe. Rooms 1 and 3 were assigned to the Heat Increase treatment and Rooms 2 and 4 to the Social Isolation treatment. Treatments started at 1330h since the majority of eggs had been laid by 0900h and an approximate 4.5-h interval occurs between egg laying and the deposition of the next eggshell. Given the number of treatments that required to be scheduled, starting at 1330h ensured all treatments were completed by approximately 1800h, allowing 1 h for researchers to finish welfare checks and for hens to eat and drink before the lights were switched off.

#### Heat increase

Heat stress is a common and much-researched stressor in poultry (e.g. Lara & Rostagno 2013). An acute heat stress was applied with a temperature increase of 11.5°C (to 30°C) for up to 2 h, since panting is observed at this temperature at a comparable level to higher temperatures and higher temperatures can result in mortality (Kang *et al.* 2020).

For the heat increase treatment, hens remained in their home room and the temperature in the room was increased to 30°C over the course of approximately 45 min (range 37–60 min), starting at 1330h. This was achieved with two additional heat lamps and the target temperature was set using the FarmX room temperature control, increasing the target temperature incrementally by 2°C. The difference in the time taken to reach target temperature was influenced by the outdoor ambient temperature. The hens were under constant observation via the remote CCTV system and/or through a one-way observation window. The treatment was stopped when over 50% of hens showed behavioural signs of the heat increase, such as panting or wing spreading. When the treatment was stopped, one researcher began to decrease the temperature in the room. Heat lamps were turned off and the target room temperature was decreased by 2°C until 18.5°C was reached.

On sampling days, once the hens had reached their stopping point, the focal bird from each pen was removed and saliva sampled as detailed below.

#### Isolation

Social isolation is a stress paradigm which has been validated for neonatal domestic chickens (Feltenstein *et al.* 2003) including with pharmacological manipulations (Warnick *et al.* 2009) and associated behaviour (Sufka *et al.* 2006). In adult chickens, isolation has been found to be associated with increased corticosterone (Ogbonna *et al.* 2022) and changes to the epigenome (Pértille *et al.* 2020).

Two pens of hens at a time were isolated by four researchers. Hens from each pen were put into transport crates and carried by two researchers down the access corridor and into the isolation room (Figure 2). Hens were then transferred to individual crates which took, on average, under two minutes. There was no food or water in the individual crates. The crates were placed along the length of the room in two rows (15 crates in each row). Hens could hear each other and had visual access to other hens, although this was restricted due to the plastic bar design of the crates (for a photograph, see Figure S1 in Supplementary material). The time of the final hen placement was noted, and a 1-h timer was started. There were five pairs of LED tube lights on the ceiling of the room, all of which remained on for the duration of isolation. The LED lights were designed for poultry sheds, each 5 ft in length, outputting 3,000 lumens (Kew Leds; UK). Our own lux measurements were variable ranging from 72.7 to 137 lux at the approximate placement of the crates (measured using an ALX-3809 light meter; ATP Instrumentation Ltd, Leics, UK).

The order of isolation was as follows: Pens 2.1 & 2.2; Pens 4.3 & 4.4; Pens 2.3 & 2.4; Pens 4.1 & 4.2. The first isolation was at ~1400h and the last at ~1700h. By alternating rooms, we left time between returning hens from the first two pens and collecting hens from the second two pens within a room. This allowed any effects of the capture and return of the first set of hens to dissipate prior to capture of the second set.

On sampling days, when the isolation time was complete, the two focal hens were removed from their crates simultaneously and saliva sampled as below. All hens were then placed back into transport crates as a group and walked back to their home pens.

### Saliva sampling

Saliva sampling was conducted with two researchers per hen. One person held the hen in their arms while the other inserted a sterile foam swab (CONSTIX® SF-2; UK) into the beak. The swab was moved around the whole mouth, making sure to swab under and over the tongue and the sides of the mouth for 120 s.

### Egg scoring

Eggs laid each day were assessed the following day. Eggs from each room were randomised into numbered baskets, such that the scorers did not know which room a set of eggs had come from. Eggs were scored by one or two researchers using the descriptors in Table 1. Each egg was given a score of 1, 2, or 3 for each attribute.

**Table 1.** Variables of eggshell quality which were manually assessed in Study 1, and the descriptors used to aid in score allocation

	Score 1	Score 2	Score 3
Texture	No texture defect, completely smooth.	Small pimples or lumps that can be felt as raised from the surface (often found at top and/or bottom). Minor localised areas of rough calcium deposits.	Large pimples or lumps Textured calcium deposits covering the majority of egg surface regardless of size of individual deposits. Orange peel texture covering the majority of egg surface. Rough patches of shell. Target shells that feel rough to the touch.
Wrinkled or creased	No wrinkles or creases, completely smooth.	Creases which appear as eggshell pores lining up. These look like small indentations only with no obvious ridges. Overall, the egg may feel smooth. If the crease is palpable, it will not be obvious to the eye.	Depressed areas flanked by ridged areas which are obvious to touch. Prominent and clearly changes the line and texture of shell surface. Can look like a cut. Corrugated eggs.
Colour	Uniform base colour. No calcium deposits and no pale shell.	Small areas of calcium splashing or white speckling. Gradient changes which remain within normal colouration for shells. Lilac eggs	Pale: as white as paper. Patchy or stripey changes in coloration or any areas of pale (white). Majority of egg surface covered with white specking, calcium splashing, calcium coating. Target shells
Shape	No misshapes	Round or long eggs if the two ends can be easily differentiated from each other. Slightly misshapen eggs e.g. slight lumps or defects in symmetry but still overall standard egg shape.	Round, long or rugby ball–shaped eggs if the two ends cannot be easily differentiated (they are almost symmetrical). Slab/flat–sided eggs Repaired eggs: Obvious repaired cracks, ridge lines around centre line. May be more easily identified by candling. Dented shells
Cracks visible	No cracks visible	Any visible crack on the shell surface.	N/A
Cracks candled	No cracks visible when candled	Finer cracks which are not visible upon observation but appear as bright lines when candled.	N/A
Shell less	Intact calcified shell present	Sometimes known as ‘rubber eggs’. The egg will be completely absent of a calcified shell layer. It will be rubbery in texture, and spongy when handled.	N/A
Thin/soft shelled	Intact calcified shell present	If the egg dents or breaks easily when handled, this will be classed as a soft or weak shell. The egg will also be tapped firmly twice on each end. If the shell dents, it will be classed as a soft or weak shell.	N/A
Pecked	Intact calcified shell present	Shells which have been pecked by a hen	N/A

Note that although all variables were scored, only texture, wrinkles and shape were eventually analysed.

Scoring eggshell characteristics by eye and touch has the potential to introduce variation via subjectivity. To combat this, researchers consulted each other when egg attributes were ambiguous or difficult to categorise. A consensus was reached and in situations where the score was borderline, researchers conservatively scored up (i.e. on the boundary of score 2 or 3, a 3 would be scored). If only one researcher was present, eggs requiring a second opinion were placed to one side to enable a second researcher to check with no indication of the initial researcher’s score.

## Statistical analysis

All data processing and analysis was conducted in R v 4.0.2 with code and data available at (https://osf.io/7cf2s/). Bayesian models were computed using the Stan programming language via the brms package (version 2.16.3), which estimates parameters using Hamiltonian Monte Carlo. Four Markov chains were run, each with a warm-up period of 1,000 iterations and 2,000 iterations used for sampling. Thinning was set to 1. Convergence was checked using the Gelman-Rubin statistic with convergence indicated by values close to 1 and less than 1.05. Model parameters were summarised by the mean and 95% highest density interval (HDI; the 95% most likely values in the distribution). Significance was inferred when the highest density interval did not contain zero.

Texture, shape and wrinkling were analysed using cumulative ordinal models (Bürkner & Vuorre 2019). Cumulative ordinal models assume a continuous latent variable underlying the ordinal categories of scores. The model estimates thresholds in place of traditional intercepts, which indicate the point on the underlying scale at which one category crosses into another. The number of thresholds is therefore one less than the number of ordinal categories, meaning our models estimated two thresholds (one threshold for score 1 crossing into 2, and a second threshold for score 2 crossing into score 3). The remaining outcome variables did not possess the variability in scores to be analysed. As the study progressed, we noted that our perception of shell colour was changing. We therefore chose not to analyse colour as we cannot be certain of the subjective reliability over time.

Predictors included in the model were: an interaction between control vs treatment days and the type of treatment (heat or isolation); a main effect of day; and a random effect of pen ID (see Table 2 for variable information).

**Table 2.** Variables and their associated details used in analyses of egg quality

Variable	Variable type
Control vs treatment days	Binary 0: control day 1: treatment day
Treatment type	Binary 0: heat treatment 1: isolation treatment
Day	Continuous 1–10: one unique number per day of study
Pen ID	Categorical 1–16: one unique number per pen

## Results

The final experimental dataset contained eggs from 234 chickens over ten days. In total, 1,338 eggs were analysed for the control period (7 days) and 595 eggs from the treatment period (3 days). The mean production percentage in the control period was 84% for the heat treatment and 87% for isolation. During the treatment phase, the mean production percentage was 88% for heat, and 89% for isolation pens. In the heat treatment, all pens showed behavioural signs of heat stress. The time taken for 50% of hens to show panting or wing spreading ranged from 28 to 46 min.

### Texture

**Figure 3.**
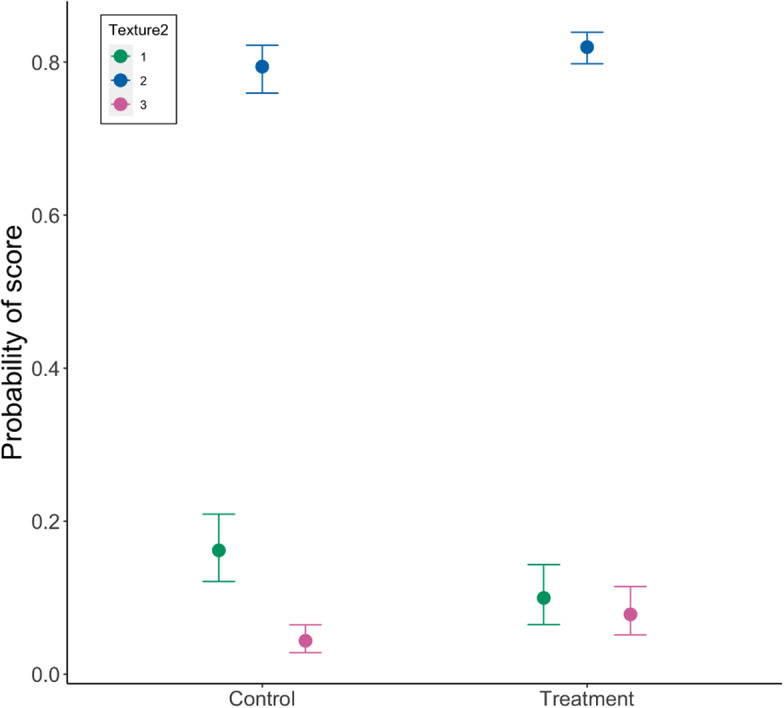
Model estimates of the impact of control vs treatment on the probability of three scores ( 1, 2, 3) of eggshell texture, where 1 is a perfect texture and 3 is a serious enough defect to quality as a Class B egg. N = 1,388 eggs in the control period, and 595 eggs in the treatment period.

Eggshell texture was significantly associated with whether eggs were laid on a control or treatment day (estimate: 0.29, 95% HDI: 0.10, 0.50), with no significant effect of any other predictor variable (Table 3). The probability of eggs scoring a perfect one for texture (no defects) was higher on control days than on treatment days, indicating an increased proportion of defects on treatment days, regardless of whether the treatment was heat or isolation (Figure 3). Variation across pens was higher than variation across days. Cross-classified random intercepts of day and Pen ID had standard deviation mean estimates of 0.05 (95% HDI: 0.00–0.14) and 0.19 (95% HDI: 0.10–0.33), respectively.

**Table 3.** Results from the cumulative ordinal model of texture defects in eggs, indicating the mean parameter estimate, the error and the lower and higher 95% highest density intervals (HDI)

	Mean	Error	Lower HDI	Higher HDI
Intercept score 1 to 2	–0.99	0.09	–1.17	–0.81
Intercept score 2 to 3	1.71	0.10	1.52	1.90
Treatment vs control	0.29	0.10	0.10	0.50
Treatment type	–0.18	0.12	–0.42	0.07
Treatment vs control × Treatment type	–0.03	0.13	–0.29	0.22

The number of eggs scored in each category was: heat, control day = 660; heat, treatment day = 297; isolation, control day = 678; isolation, treatment day = 298. Note that results are presented on the latent, standard normal distribution scale.

### Shape

**Figure 4.**
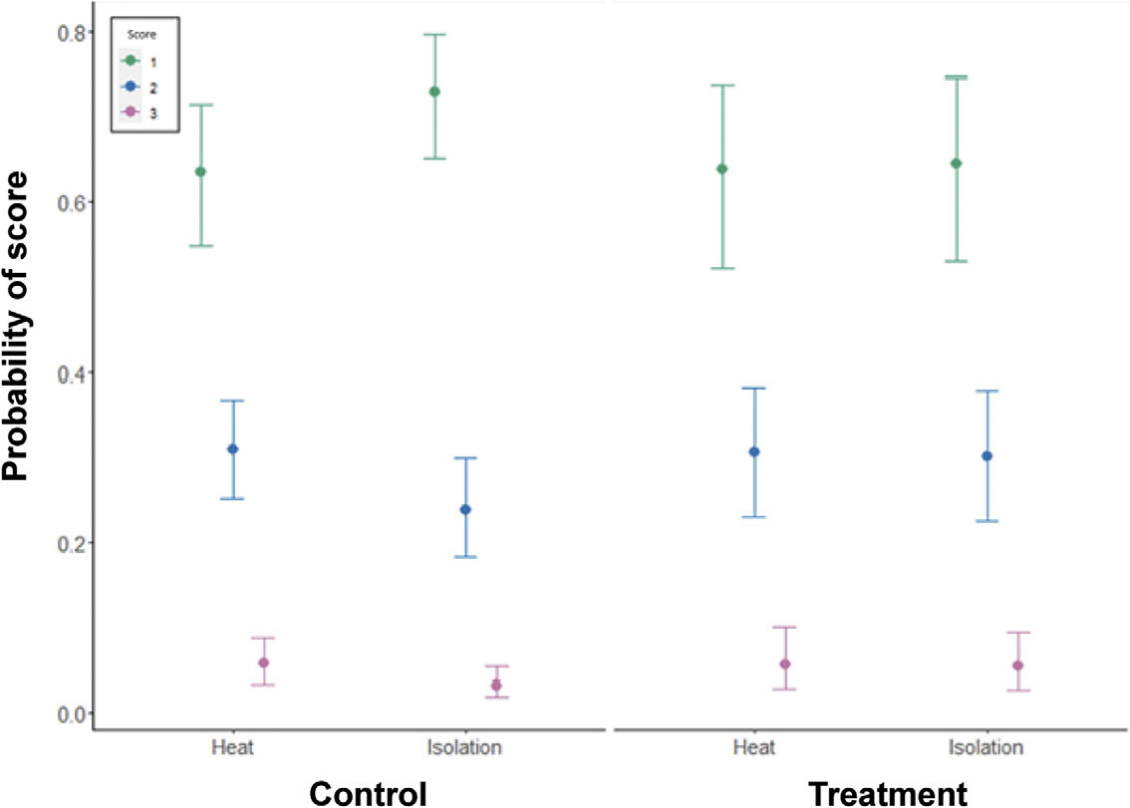
Model estimates of the impact of control vs treatment and treatment type (heat or isolation) on the probability of three scores (1, 2, 3) of egg shape, where 1 is a perfect shape and 3 is a serious enough defect to qualify as a Class B egg. The number of eggs scored in each category was: heat, control day = 660; heat, treatment day = 297; isolation, control day = 678; isolation, treatment day = 298.

Egg shape was significantly associated with an interaction between treatment vs control days and treatment type (estimate: 0.25, 95% HDI: 0.01–0.48; Table 4). The probability of eggs scoring a one (perfect eggs) was higher in the isolation group but only during the control period (Figure 4). Variation across pens was similar to variation across days. Cross-classified random intercepts of day and Pen ID had standard deviation mean estimates of 0.17 (95% HDI: 0.06–0.36) and 0.20 (95% HDI: 0.11–0.34), respectively.

**Table 4.** Results from the cumulative ordinal model of shape defects in eggs, indicating the mean parameter estimate, the error and the lower and higher 95% highest density intervals (HDI)

	Mean	Error	Lower HDI	Higher HDI
Intercept score 1 to 2	0.34	0.11	0.13	0.56
Intercept score 2 to 3	1.58	0.12	1.36	1.82
Treatment vs control	–0.01	0.15	–0.31	0.29
Treatment type	–0.27	0.13	–0.52	–0.01
Treatment vs control × Treatment type	0.25	0.12	0.01	0.48

The number of eggs scored in each category was: heat, control day = 660; heat, treatment day = 297; isolation, control day = 678; isolation, treatment day = 298. Note that results are presented on the latent, standard normal distribution scale.

### Wrinkles

**Figure 5.**
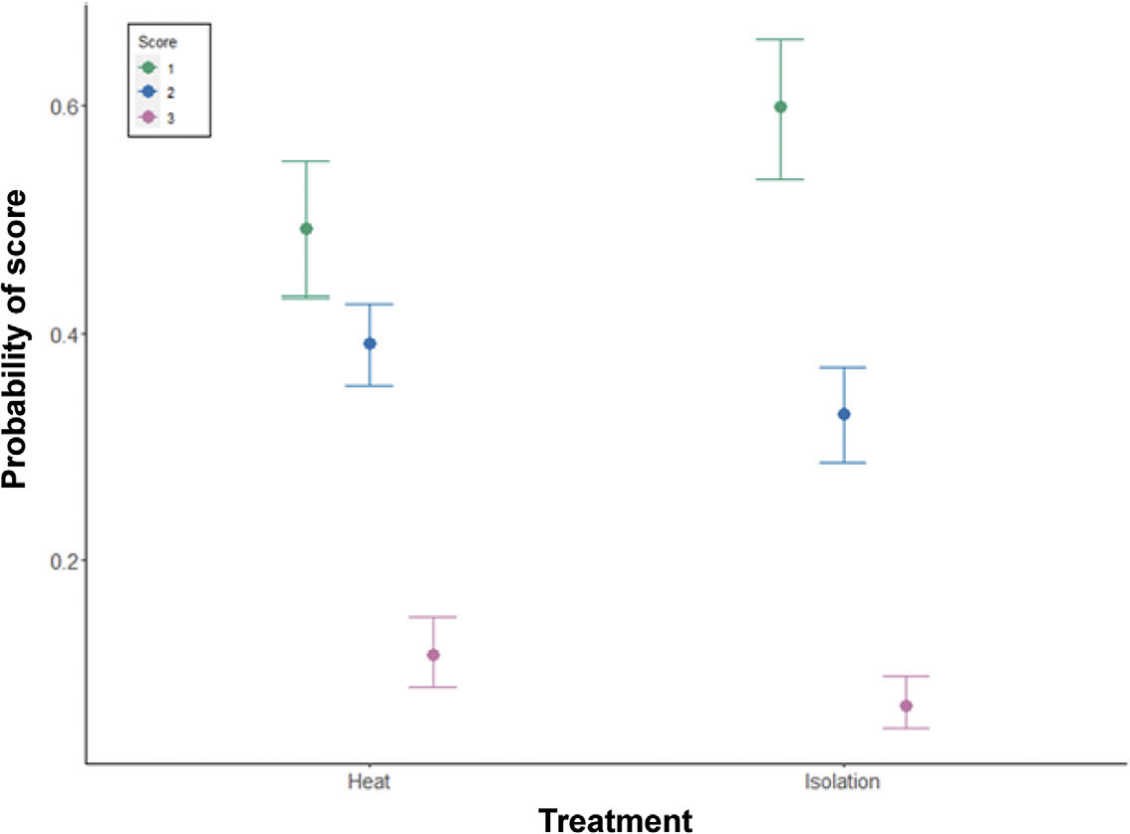
Model estimates of the impact of treatment type (heat or isolation) on the probability of three scores (1, 2, 3) of egg wrinkling, where 1 has no wrinkles and 3 is a serious enough defect to qualify as a Class B egg. Nine hundred and fifty-seven eggs were scored in the heat treatment and 976 eggs in the isolation treatment.

Wrinkles on the eggs’ surface was significantly associated with treatment type, with a higher proportion of the score 1 for hens undergoing the isolation treatments (Figure 5; estimate: –0.27, 95% HDI: –0.47– –0.07). There were no significant associations with any other predictor variables (Table 5). Variation across pens was higher than variation across days. Cross-classified random intercepts of day and Pen ID had standard deviation mean estimates of 0.08 (95% HDI: 0.00–0.21) and 0.14 (95% HDI: 0.05–0.25), respectively.

**Table 5.** Results from the cumulative ordinal model of wrinkle defects in eggs, indicating the mean parameter estimate, the error and the lower and higher 95% highest density intervals (HDI)

	Mean	Error	Lower HDI	Higher HDI
Intercept score 1 to 2	–0.02	0.08	–0.18	0.14
Intercept score 2 to 3	1.19	0.09	1.02	1.36
Treatment vs control	–0.06	0.11	–0.27	0.15
Treatment type	–0.27	0.10	–0.47	–0.07
Treatment vs control × Treatment type	0.11	0.12	–0.12	0.34

The number of eggs scored in each category was: heat, control day = 660; heat, treatment day = 297; isolation, control day = 678; isolation, treatment day = 298. Note that results are presented on the latent, standard normal distribution scale.

## Study 2

## Materials and methods

Eggs were collected from five flocks across four farms before and after the enforced indoor housing was in place. A previously established relationship with these farms enabled eggs to be gathered at relatively short notice following the announcement of enforced indoor housing. Four flocks were Shaver Browns, and one was Lohmann Classic, all housed on flat deck systems. The ages of the flocks ranged from 30–66 weeks and flock sizes were 3,000, 12,000 and three flocks of 16,000 at placement. The number of eggs collected from each flock for scoring was calculated (5% of the flock size) and the same number of eggs were collected for one day before confinement and one day after confinement, i.e. if confinement commenced on day 0, eggs were collected on day –1 and day +1. The egg-packing factory was asked to hold back the required number of eggs before eggs were graded and researchers were not involved in egg selection. These eggs were then manually scored for texture (as the most promising indicator of stress) using the same method as in Study 1. There were five scorers of the commercial eggs, two of whom had scored eggs in Study 1.

**Figure 6.**
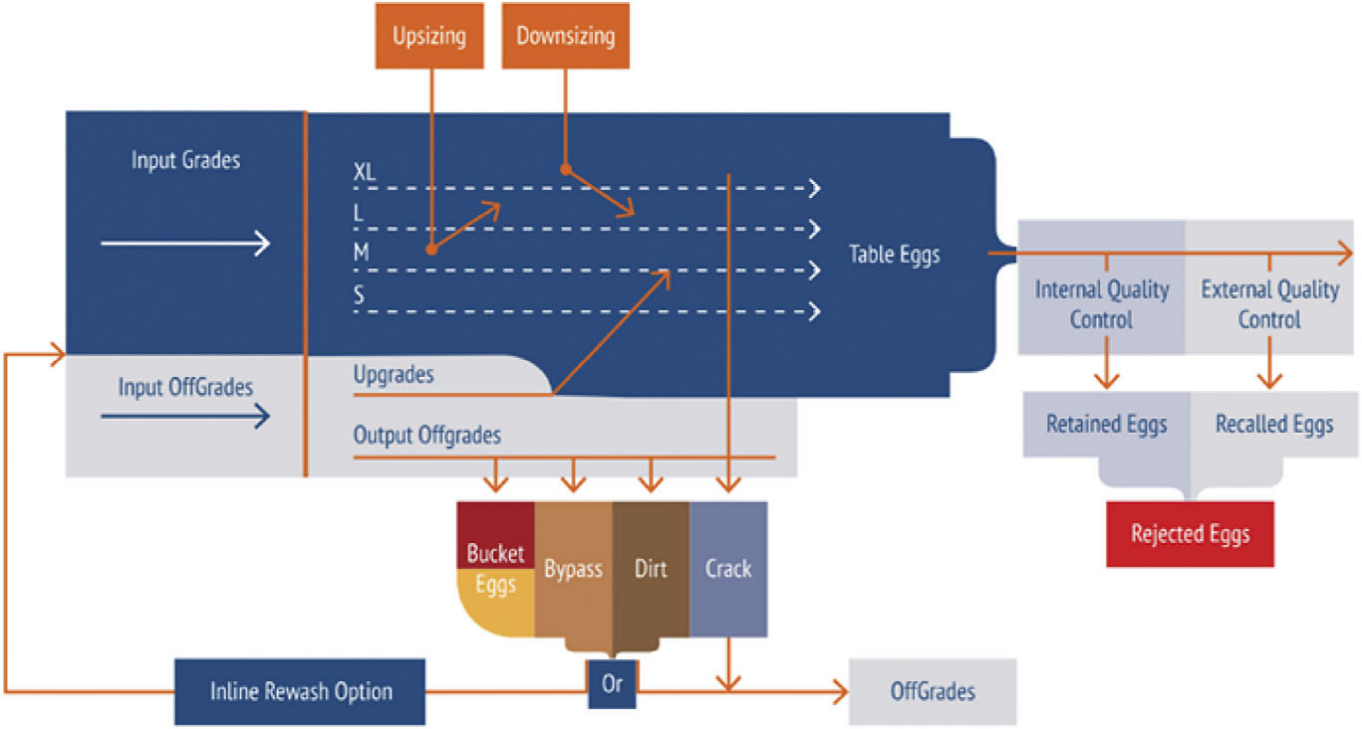
Diagram showing the process by which eggs from laying hens go through a commercial grading machine. Image owned by, and used with permission from, iMOBA.

Flock-level data were also collected for these five flocks from iMOBA. iMOBA is an online portal through which data collected from egg grading/packing machines (the process of which can be seen in Figure 6) can be accessed to give a large amount of data on egg production and quality with little gathering effort. Every egg on the production line passes through one of these grading/packing machines allowing for the data to represent the entire flock. This system gives each egg a score for colour, size, shell strength and offgrades. Colour is measured by brownness on a scale of 0–9 (0: white, 9: dark brown), based on light reflectivity. Size is determined by egg weight and has nine divisions from XS to XL. Shell strength is measured on a scale of 1–10 (1: weakest, 10: strongest). This is achieved acoustically via eight probes that measure the egg at multiple points on the shell and uses an average of the five best measurements (deviating least from the mean). Each probe has a metal ball in a magnetic field. Shell strength is inferred from the contact time of the steel ball with the shell using sound generated by the ball hitting the egg to determine the contact time. A shorter contact time indicates a stronger shell. There is also a score for offgrades (i.e. leaker, blood, crack, dirt, and other) which is presented as a proportion of the total number of eggs to have that specific deformity. Blood and different types of dirt are detected using prism RGB cameras which split the red, green, and blue light signals, allowing for differentiation of dirt marks. Cracks are determined using the same probes as for shell strength, but instead of contact time, the number of pulses recorded by the probe is used to detect cracks. When the metal balls hit the eggshell, they make a sound that is recorded. When the sound is above a volume threshold this registers as a pulse. This pulse count is a measure of quality and is translated to a scale of 1–30 whereby 1 is a very fine crack and 30 is a leaker.

In addition to the five flocks in which we compared texture and commercial grading, we also carried out an exploratory analysis of commercial grading data for an expanded number of farms. Producers were contacted from a pool who supply the partner egg-packing company. We restricted our producers to those in England, as other parts of the UK differed in biosecurity zones and enforced indoor housing duration. Producers who consented to their egg data being used were included in the expanded dataset which amounted to 40 flocks from 23 farms. Three flocks were excluded as they produced blue eggs.

## Statistical analysis

We used cumulative ordinal models as described in Study 1 to analyse texture. The enforced indoor housing was included as a binary fixed effect with two levels (before or after). Flock ID was also included as a fixed effect, rather than as random effect, as there were only five flocks. The youngest flock was used as a reference category in the model.

To explore whether texture (as a promising egg quality measure from Study 1) could be correlated with measures already collected by the egg-packing factory, we conducted paired Spearman’s correlations. The correlations were between the average texture measure per flock, which was manually scored, and a flock level summary of each of the measures available from the iMOBA system. These were: Average shell strength; Average shell colour; Proportion of leakers; Proportion of blood; Proportion of cracked eggs; Proportion of dirty eggs; Proportion of other defects; Total proportion all defects; Average egg size. Averages were created from ordinal scores which, although not ideal, provided a single per farm measure to allow comparison. Finally, we selected measures from the egg-packing factory which correlated highly (0.8+) with texture and visually explored variation in these before and after the enforced indoor housing in the expanded dataset of 37 flocks.

## Results

We aimed to score 5% of eggs for each flock and were close to achieving this with a range of 4.9–5.5% (150–795) of eggs laid per day scored for each farm. Some eggs could not be scored for texture and had to be excluded because they were dirty: 33 were excluded before the enforced indoor housing and 36 after. In total, 6,201 eggs were scored: 3,087 eggs were scored before, and 3,114 eggs scored for after the enforced indoor housing. The following numbers of eggs achieved each score: Score 1: 2,253 eggs (36.3%); Score 2: 3,626 eggs (58.5%); Score 3: 322 (5.2%).

**Figure 7.**
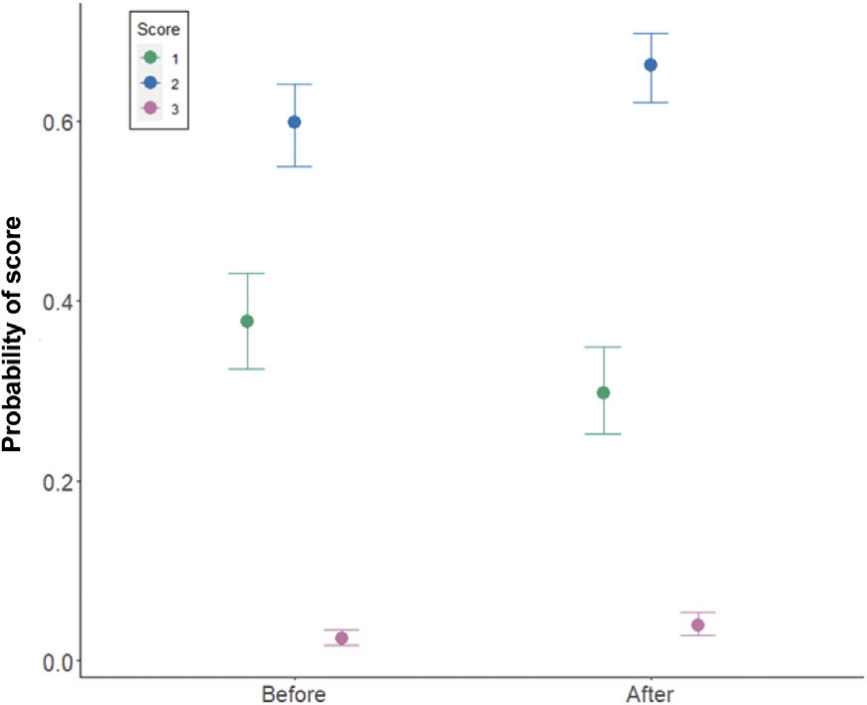
Model estimates of the impact of before or after the enforced indoor housing on the probability of three scores (1, 2, 3) of egg texture where 1 is a perfect texture and 3 is a serious enough defect to quality as a Class B egg. Three thousand and eighty-seven eggs were scored before enforced indoor housing, and 3,114 eggs scored after the enforced indoor housing.

**Figure 8.**
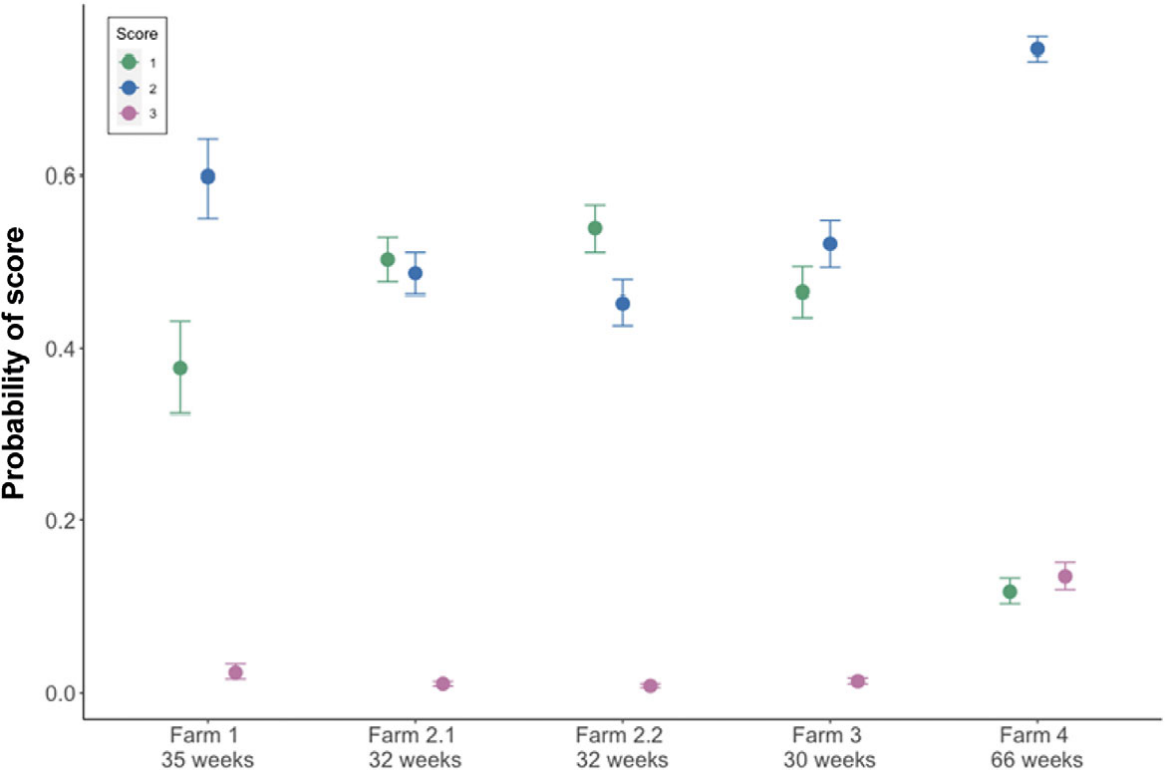
Model estimates of farm differences on the probability of three scores of egg texture, where 1 is a perfect egg and 3 is a serious enough defect to qualify as a Class B egg. ID is anonymised but provided as a Farm number followed by a flock number within Farm, where data were collected from more than one flock per farm. Age of the flocks at the time of egg collection is provided for context. The number of eggs scored per farm was as follows: Farm 1 = 300; Farm 2.1 = 1,557; Farm 2.2 = 1,557; Farm 3 = 1,200; Farm 4 = 1,587.

Eggshell texture was significantly associated with whether eggs were laid before or after the enforced indoor housing (estimate: 0.22, 95% HDI: 0.16, 0.28; see Table 6). The probability of eggs scoring 1 for texture (no defects) was higher before the enforced indoor housing than after (Figure 7). Variation across farms was modelled as a fixed effect as there were only five farms and there was variation between farms in the probability of laying a perfect egg which is plotted against age in Figure 8 and shown in Table 6.

**Table 6.** Results from the cumulative ordinal model of texture defects in eggs on commercial farms before and after enforced indoor housing of chickens as a biosecurity measure, indicating the mean parameter estimate, the error and the lower and higher 95% highest density intervals (HDI)

	Mean	Error	Lower HDI	Higher HDI
Intercept score 1 to 2	–0.31	0.07	–0.44	–0.16
Intercept score 2 to 3	1.98	0.08	1.83	2.13
Before vs After	0.22	0.03	0.16	0.28
Farm 2.1	–0.32	0.07	–0.46	–0.17
Farm 2.2	–0.41	0.07	–0.55	–0.26
Farm 3	–0.22	0.08	–0.37	–0.07
Farm 4	0.88	0.08	0.73	1.03

3087 eggs were scored before enforced indoor housing, and 3114 eggs scored after the enforced indoor housing. Note that results are presented on the latent, standard normal distribution scale.

There were a number of strong correlations between the manual score for texture and the measures of egg quality from matched egg-packing data. The strongest correlations were with Average Shell Strength, and Average Shell Colour (see Table 7). We explored these measures of egg quality in a larger dataset of farms comparing before and after the housing order enforced indoor housing.

**Table 7.** Results from the correlations between the manual score of texture per farm and average automated measures of egg quality per farm

	Correlation
Average shell strength	–0.903
Average shell colour	–0.758
Proportion of leakers	0.539
Proportion of blood	–0.413
Proportion of cracked eggs	.673
Proportion of dirty eggs	.758
Proportion of other defects	–0.60
Average egg size	0.188

**Figure 9.**
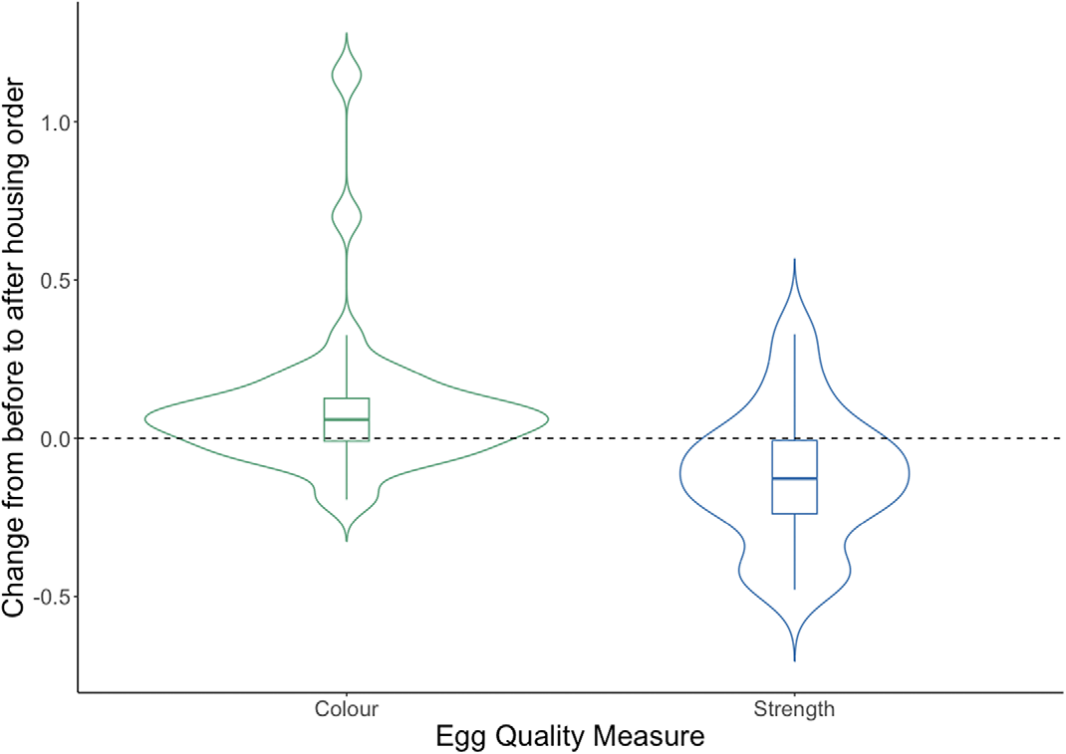
Distribution of change, from before to after the enforced indoor housing, in average scores for two commercially collected automated measures of egg quality from 37 flocks of laying hens Details of colour and strength scoring can be found in the *Materials and methods* for Study 2.

We were able to gather commercial egg quality data from a further 40 flocks but excluded data from three as these produced blue eggs. Overall, there were consistent changes in average shell colour (darker by 0.09) and average shell strength decreased by 0.12 (see Figure 9, and Figures S2 and S3 in the Supplementary material for distributions). After the enforced indoor housing was put into place, the colour of the eggs became darker (increased) in 26/37 farms, the average size increased in 26/37 farms and shell strength decreased in 29/37 farms. These changes were not apparent from looking at the overall proportion of off-grades which increased on 15 farms and decreased in 22 farms.

## Discussion

In this study, we were able to use a manipulation of heat stress which resulted in wing spreading, lethargy (observed as decreases in activity) and panting (H1). We found that this subtle heat stressor, and an isolation stressor, were associated with changes in egg quality parameters (as predicted by H2). Hens which had experienced either a heat or isolation stressor in the previous day were less likely to lay a smooth textured egg than during a control period prior to experience of the stressors. This suggests that texture could be a domain-general indicator of stress rather than changes being related to a specific stressor type. There were also some interesting associations of shape and wrinkles with the treatments, but there were no clear differences between control vs treatment days, potentially evidencing relatively inflexible individual variation in some egg traits. The most promising measure of eggshell quality from Study 1, texture, also increased in commercial flocks in immediate (day 1) response to a presumed stressor, the biosecurity enforced indoor housing of free-range chickens in the UK for Avian Influenza (H3). Finally, we identified two measures of egg quality from the egg-packing factory which were highly correlated with manually scored texture: shell strength and shell colour. Before the enforced indoor housing, shells appeared to be stronger, and eggs were lighter in colour. From these findings we would cautiously hypothesise that shell strength and shell colour are promising proxy measures of stress in free-range commercial chickens, though below we expand upon the complexities and intricacies which must be considered in relation to these parameters.

While shell colour and shell strength also varied with the commercial stressor, texture specifically could be a useful additional measure for egg-packing factories to incorporate, as here this been experimentally and commercially tested and found to be responsive to stressors. Texture was the most promising indicator of stress from this study as fewer smooth eggs were found in response to three different stressors (heat, isolation, and the enforced indoor housing) in experimental and commercial conditions. Changes to eggshell texture in response to stress are expected based on previous literature outlined in the *Introduction*, suggesting that stress causes a delay in laying (Sykes 1955; Hughes & Gilbert 1984; Hughes et al. 1986; Reynard & Savory 1999) and during this time additional calcium can be deposited on the shell. Biologically, it is important for birds to be able to respond to environmental and internal conditions to lay eggs at a more optimal time and stress hormones are an important mechanism (Goutte *et al.* 2011). Whilst time of laying could be a useful indicator of stress in individuals, measuring this on a commercial scale would be challenging, so indicators of that delay have great potential as a stress indicator. Birds in Study 1 were 59–63 weeks of age which falls after peak egg production, and the impact of age on eggshell microstructure has been previously documented (Benavides-Reyes et al. 2021). Therefore, external eggshell traits need to be further characterised across the laying cycle to assess the generalisability of the results.

It is important to acknowledge that egg texture was not always scored ‘blind’ which allows for experimenter bias (Holman *et al.* 2015). Whilst blinding was possible during Study 1, in Study 2 it was known to scorers whether eggs were produced before or after the enforced indoor housing based on the date. However, the eggs which were manually scored for texture correlated highly with automated measures of egg quality from the factory. Since the automated measures could not be biased, it seems unlikely that bias in scoring alone explains our results. Any future studies in this area could combine the methods across our two studies to measure texture and shell strength from the same eggs, helping to further elucidate the relationship. In Study 1, we did not have the planned number of treatment days due to extreme and disruptive weather conditions causing us to end the experiment earlier than planned. In addition, we acknowledge that the saliva sampling of focal birds may have itself been a stressor. However, given that only one bird per pen underwent this treatment, we think it unlikely to have impacted the egg-scoring results. Due to these limitations, we suggest that our results on egg texture be viewed with cautious optimism given the shared findings across studies presented here.

The results from Study 1 for shell wrinkles and egg shape did not fit with expectations. For shape, there were more perfect eggs in the hens in the isolation treatment pens, compared to the heat treatment pens, during the control days. As there had been no experimentally induced differences during the control period, the most likely explanation is individual variation in egg shape and chance allocation of individuals with different-shaped eggs across treatments. Indeed, many qualities of eggs appear to be consistent within an individual and differ between hens (Cheng & Ning 2023). It is notable that there appear to be fewer perfect-shaped eggs after the isolation stressor, compared to the control for this group, and this might be explained by an increase in handling which has previously been associated with changes in egg shape (Hughes & Black 1976). For wrinkles of eggs, there were more found in the hens in the isolation treatment, compared to the heat stress hens, but there was no effect of whether the birds had experienced that stressor (treatment) or whether it was a control day. Again, the most likely explanation for this is that assignment of the hens was not random with respect to the propensity to wrinkle between treatments. Future experiments on egg quality measures could consider randomising hens according to eggshell defects, but this would require an initial phase of scoring of eggs for individual hens which could be achieved using coloured dyes (as in Rufener *et al.* 2019) or smart nest-boxes (as in Chien & Chen 2018). We discounted our scores of eggshell colour, as we could not be confident in the subjective scoring of colour over time. The difficulty in manually assessing eggshell colour and the subtle differences in colour abnormalities was also noted by Hughes et al. (1986). In any future studies we conduct, a combination of subjective scores of heterogenous traits (such as calcium splashing) and objective measures of base shell colour will be used.

In Study 2 we found that, on average, eggs from 37 flocks got darker and weaker after hens’ access to the range was withdrawn. Withdrawing access to the range was a presumed stressor due to withdrawal of a valued resource, however, we did not quantify range use before the enforced indoor housing and so cannot know how many birds were directly impacted by prohibited access to a previously used area. A review by Pettersson et al. (2016) reported between 10–50% of free-range flocks to be outside at any one time. An in-depth study by Richards *et al.* (2011) tagged 10% of four flocks and found ~80% of the tagged birds accessed the pop holes. We therefore think it a fair assumption that enforced indoor housing impacted a sufficient proportion of the flock to detect changes in stress, but we note that stronger conclusions could be drawn with more egg data on either side of enforced indoor housing to account for any daily fluctuations in shell characteristics.

In addition to withdrawn use of the range, there may also be other impacts of the enforced indoor housing. Access to the range is typically through opening of pop holes and keeping these shut during the enforced indoor housing could impact the temperature, lighting, ventilation, circulation of gases and the stocking density experienced during hours when pop holes would normally be open. Diet could also be impacted, although the extent to which hens feed from foraging on the range is not known (Miao *et al.* 2005). Previously, shell strength has been found to be weakened in response to heat stress (Mignon-Grasteau *et al.* 2015), but effects on strength have not been found in response to feeding glucocorticoids (Kim *et al.* 2015; Oluwagbenga *et al.* 2023). It is pertinent to note that Dawkins *et al.* (2004) found no relationship between shell strength and hen preference for an enriched versus barren environment and, accordingly, urged caution in relying upon eggshell characteristics without considering hen choice. As data on shell strength were exploratory, it would be useful for further research to elucidate the relationship between shell strength and stress, while also considering any important hen preferences. Previous research has found that brown eggs get lighter following a stressor due to additional calcium deposits associated with delayed oviposition (for reviews, see Hughes et al. 1986 and Samiullah et al. 2015). This is contrary to our findings; however, our results may have been influenced by a change in hens’ exposure to sunlight after the enforced indoor housing. There is some evidence that more time spent outdoors contributes to paler eggs, though this is flock-dependent (Icken et al. 2011) and the mechanism is unclear. Previous assumptions linked pale shells to increased vitamin D (caused by increased sunlight), however this relationship has been shown experimentally to be weak and may be more relevant on a longer time-scale than the short-term effects explored in our study (Roberts *et al.* 2014).

Within commercial data from the egg-packing factory, such as that accessed here from the iMOBA system, there is a wealth of information for researchers to explore. Moreover, there is great potential to increase sensitivity of the markers discussed above by understanding and integrating across different shell characteristics. For example, we observed a stronger correlation between shell texture with strength than cracks, which may be expected if proportion of cracked eggs conflates shell weakness with more random, post-lay events. While strength may be the better stand-alone indicator, the relative explanatory power of ‘cracks’ could pinpoint behavioural (e.g. startling) or structural (e.g. substrate) risk factors for post-lay egg loss. Our data exploration was limited to flock average measures, but it could be that the distribution of eggs across the different scores (egg colour and strength are recorded in ten categories) is more informative of hen health or welfare. Any approaches which used eggshell quality as a potential stress indicator would require an understanding of what is normal for the age, strain, and system. Many qualities of eggs are impacted by nutrition and disease factors (Cheng & Ning 2023). Thus, future research could explore whether eggs may be used in combination with other indicators to support digital diagnosis of health and welfare problems in free-range laying hens.

### Animal welfare implications

The results of the studies presented here indicate that qualities of laying hen eggshells are sensitive to stressors. Egg texture, in particular, appears to be a measure which responds to a variety of stressors, including in commercial settings. Egg texture can be measured non-invasively without any requirement for contact with hens and can be measured for large numbers of individuals. Scoring of eggs could be used as a complementary measure to hen welfare audits which do require disruption to a flock, biosecurity risks and typically only measure a small proportion of individuals within a flock. Eggshell measures could be used to provide stockpersons with up-to-date information on hen stress which may help them to understand and mitigate stressors for their flock. Using eggs to detect stressors encountered over time could allow researchers a better understanding of cumulative stress. We believe that eggshells have great potential to contribute to on-farm welfare assessment, management and welfare science.

## Supporting information

Gray et al. supplementary material Gray et al. supplementary material

## Supplementary material

The supplementary material for this article can be found at http://doi.org/10.1017/awf.2024.46.
