# Supplementary material for: Can eggshells indicate stressor exposure in free-range laying hens?
Source: Anim Welf. 2024 Nov 20;33:e53. doi: 10.1017/awf.2024.46 (PMC11655278; doi:10.1017/awf.2024.46)
Supplement: Gray et al. supplementary material [file S0962728624000460sup001.pdf]

Supplementary material

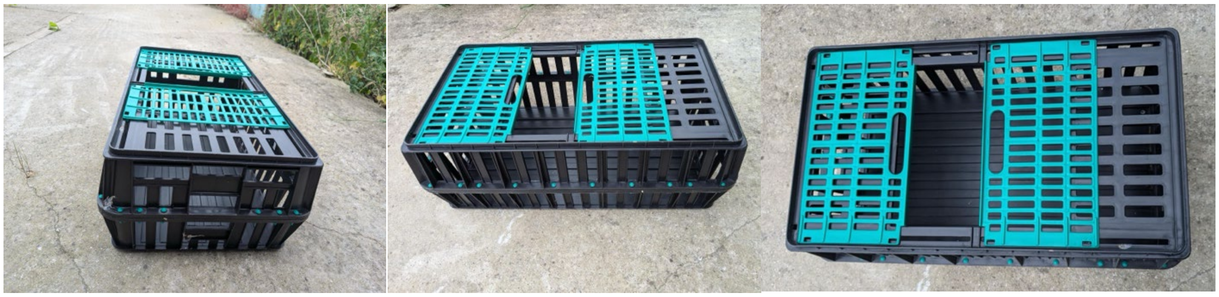

Figure S1. Photographs of transport crates used in the isolation treatment.

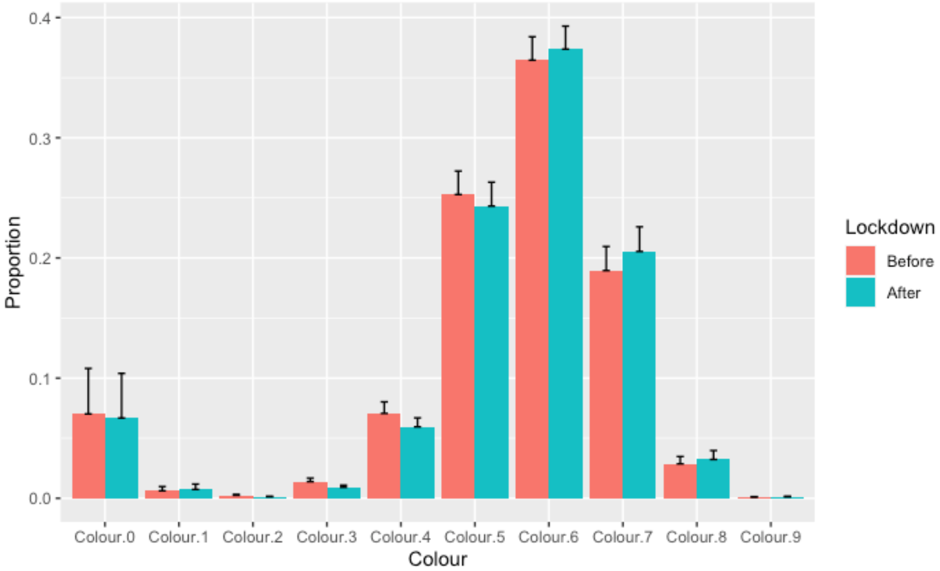

Figure S2. Distributions of colour of eggshells recorded from five flocks before and after the housing order where access to range was withdrawn.

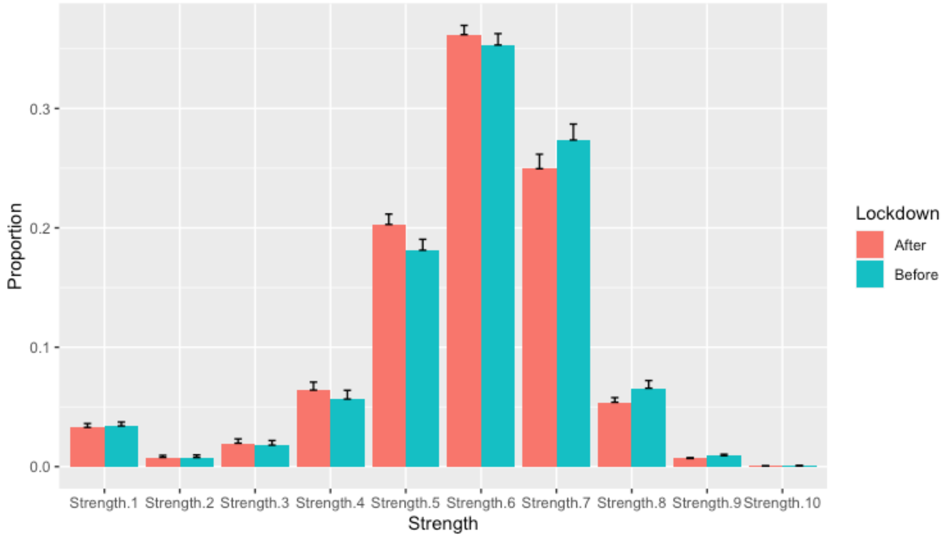

Figure S3. Distributions of eggshell strength recorded from five flocks before and after the housing order where access to range was withdrawn.
